# Necrotizing Pneumonia: A Rare Outcome of a Rare Complication

**DOI:** 10.7759/cureus.51527

**Published:** 2024-01-02

**Authors:** Francisco Dá Mesquita Faustino, Catarina Morgado, Inês Palmares, Joana Ferrão, Paulo Freitas

**Affiliations:** 1 Critical Care Medicine, Hospital Prof. Doutor Fernando da Fonseca, Amadora, PRT; 2 Critical Care Unit, Hospital Prof. Doutor Fernando da Fonseca, Amadora, PRT; 3 Intensive Care Unit, Hospital Prof. Doutor Fernando da Fonseca, Amadora, PRT

**Keywords:** critical care, invasive mechanical ventilation, ventilation, sepsis and shock physiology, necrotizing pneumonia

## Abstract

Necrotizing pneumonia is a rare complication of community-acquired pneumonia, characterized by lung parenchymal destruction in affected areas. It has been sporadically documented over the years, mostly with insidious progression and a higher incidence among individuals with risk factors. Its diagnosis relies on clinical, analytical, and imaging data, and the treatment encompasses medical and surgical measures. The persistently high morbidity and mortality result from delayed diagnosis and the intricate therapeutic approach. In this report, the authors describe an unusual case of necrotizing pneumonia in a patient without risk factors.

## Introduction

Necrotizing pneumonia (NP) is a severe community-acquired pneumonia (CAP) complication. It is characterized by lung tissue liquefaction, leading to lung cavitation. The availability of thoracic computed tomography (CT) has led to increased diagnoses, suggesting it may have been underreported in the past, with significant consequences for affected patients. The most common causative agent is Streptococcus pneumoniae, but others, including different Streptococcus species, Staphylococcus aureus, Pseudomonas aeruginosa, and even anaerobic microorganisms, such as Fusobacterium, have been identified [1]. The destruction of lung tissue results from a multifactorial process influenced by microorganisms and host factors [1,2].

Clinically, individuals commonly experience fever, productive cough, shortness of breath, and respiratory failure. On the analytical front, findings include anemia, leukocytosis with neutrophilia, significant elevation in C-reactive protein (CRP) and procalcitonin (PCT), as well as hypoalbuminemia [1,3]. The common microbiological tests used are culture examination of sputum and bronchial secretions, bronchoalveolar lavage when bronchoscopy is performed, pleural fluid analysis in cases of pleural effusion, and blood cultures. The gold standard for diagnosing NP is chest CT, although chest radiography serves as the initial and highly valuable diagnostic tool for managing these patients [3].

Medical treatment involves empiric broad-spectrum antibiotics with coverage for the coverage for the primary causative agents [3,4]. If a microbiological test reveals a pathogen, the antibiotic therapy must be directed at it. In cases of pleural effusion, pleural drainage is recommended [4]. Surgical intervention is indicated when medical treatment proves ineffective or when complications, such as a bronchopulmonary fistula leading to persistent pneumothorax and/or loculated empyema, arise [4].

## Case presentation

**Figure 1:**
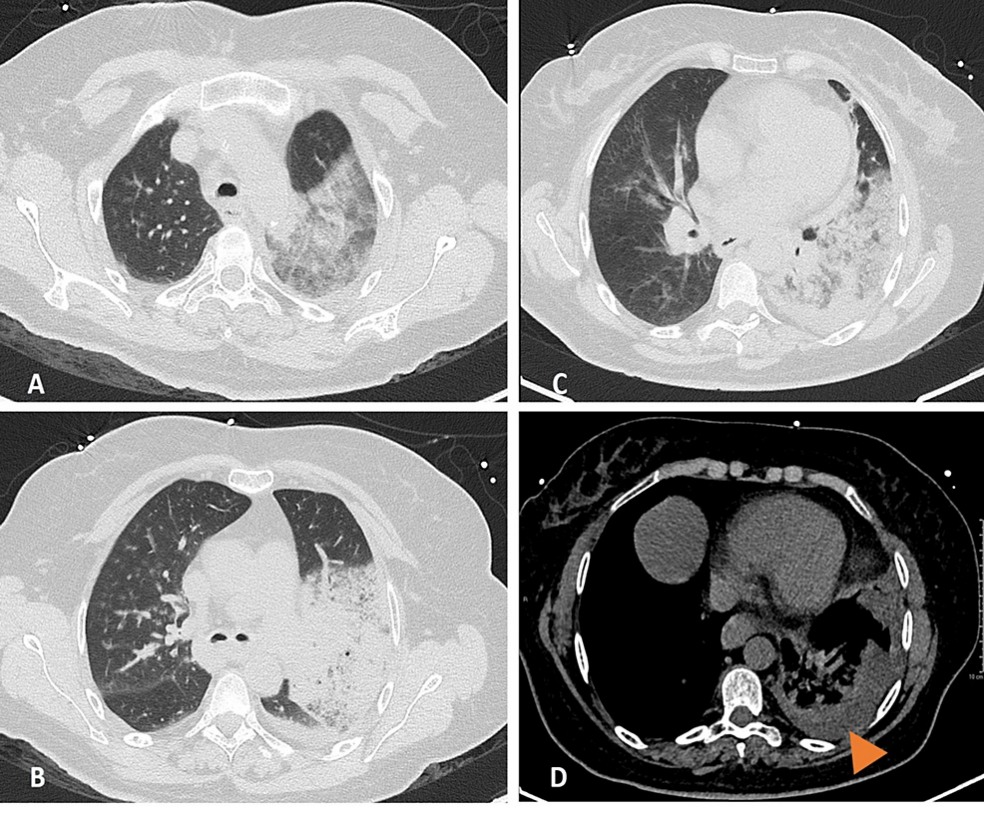
Chest computed tomography performed on the day of admission. Axial unenhanced computed tomography images in the lung window at the apex (A), mid-lung (B), and base (C) demonstrate areas of consolidation in the left upper and lower lobes, with air bronchogram and surrounding ground glass opacities. Axial unenhanced computed tomography image in a mediastinal window at the base (D) shows a small left pleural effusion (arrowhead).

A 71-year-old female, leukodermic, with a medical history of essential arterial hypertension, dyslipidemia, peripheral venous insufficiency, and degenerative osteoarticular spine disease, was a nonsmoker and a nonalcoholic. She presented at the emergency department (ED) with respiratory distress, productive cough, and pleuritic chest pain for approximately 48 hours, with no reported fever or other symptoms. Upon admission, the patient was conscious, oriented, and cooperative, with a Glasgow Coma Scale (GCS) score 15. Her vital signs included a blood pressure of 95/57 mmHg, a heart rate of 105 bpm, a respiratory rate of 23 cpm, and peripheral oxygen saturation of 91% with O2 administered at 4 L/min via nasal cannula. Lung auscultation revealed reduced breath sounds on the left and scattered subcrepitating sounds. Gas analysis (under an inspiratory fraction of O2 31%) indicated type 1 respiratory failure: pH 7.34, pCO_2_ 39, pO_2_ 65, HCO_3_ 20, SatO_2_ 96%, and lactate 2.1 mmol/L. The laboratory results are presented in Table 1. Chest X-ray showed decreased transparency in the middle third of the left lung. Therefore, she was referred for a thoracic CT scan, which showed a parenchymal consolidation in the upper lobe of the left lung extending to the lingula, accompanied by an air bronchogram (Figure 1).

**Table 1:** Laboratory test results

Laboratory Test	Actual Result	Normal Range
White blood cells (WBC)	2.0 x 10^9^/L	4-10 x 10^9^/L
Neutrophils	1.0 x 10^9^/L	2-8 x 10^9^/L
Lymphocytes	0.2 x 10^9^/L	1-4 x 10^9^/L
Creatinine	2.2 mg/dL	0.8-1.3 mg/dL
Blood Urea Nitrogen (BUN)	65.0 mmol/L	1.2-3 mmol/L
C-reactive protein (CRP)	22.0 mg/L	< 5 mg/L
Procalcitonin (PCT)	42.0 ng/mL	< 0.1 ng/mL
Albumin	30.5 g/L	35-50 g/L

In the ED, empirical antibiotic therapy with ceftriaxone and azithromycin was initiated after collecting bronchial secretions and blood samples for culture. During her ED stay, an episode of hemoptysis occurred, leading to significant ventilatory and hemodynamic consequences. This necessitated orotracheal intubation, invasive mechanical ventilation, and the initiation of vasoactive support. In this critical condition, the patient was transferred to the intensive care unit (ICU) with a preliminary diagnosis of septic shock stemming from CAP with respiratory and renal dysfunction. Upon admission to the ICU, a bronchoscopy was performed, revealing active bleeding in the left bronchial tree, which required antifibrinolytic treatment involving aminocaproic acid in conjunction with adrenaline and cold saline. After hemorrhage control, bronchial secretions were collected, and bronchoalveolar lavage was conducted on the upper segment of the lingula. The respiratory virus panel (respiratory syncytial virus, influenza virus, and SARS-CoV-2), antigenuria (S. pneumoniae and L. pneumophila), and hemocultures were negative. Cultures of bronchial secretions and bronchoalveolar lavage revealed the presence of S. pyogenes. An antibiotic sensitivity test confirmed that penicillin was an appropriate choice for targeted antibiotic therapy after completing a 5-day course of ceftriaxone and azithromycin.

**Figure 2:**
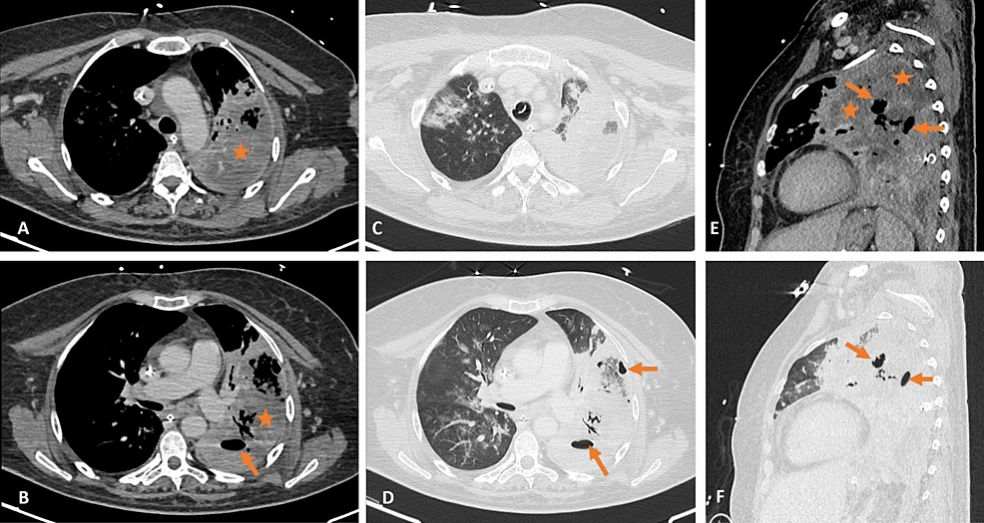
Chest computed tomography performed 11 days after hospitalization. Axial enhanced computed tomography images in a mediastinal window at the apex (A) and mid-lung (B) demonstrate heterogeneously enhancing left lung consolidation (asterisks), suggestive of pulmonary necrosis, with air foci (arrows) reflecting abscess formation. Axial enhanced computed tomography images in the lung window at the apex (C) and mid-lung (D) reveal increased left lung consolidation and air foci (arrows), in addition to right lung infiltrates. Sagittal reformatted images in mediastinal (E) and lung (F) windows depict the same findings.

During her hospitalization, the patient remained afebrile, but her acute phase parameters worsened, with progressive difficulty in maintaining protective ventilation. This necessitated adjusting her volume control ventilation strategy, permitting respiratory acidosis due to 6 mL/kg tidal volumes with lower invasive mechanical ventilation driving pressures of >15 cmH_2_O. On the 10th day of hospitalization, a repeat chest CT scan revealed a reduced volume of the left lung parenchyma, characterized by a consolidation with hypodense and hypocapturing confluent areas, accompanied by gas bubbles, indicating necrosis. There was also pleural effusion in the internal segment of the left basal pyramid, resulting in passive atelectasis of the adjacent parenchyma (Figure 2). Therefore, clindamycin was added to the ongoing antibiotic therapy.

**Figure 3:**
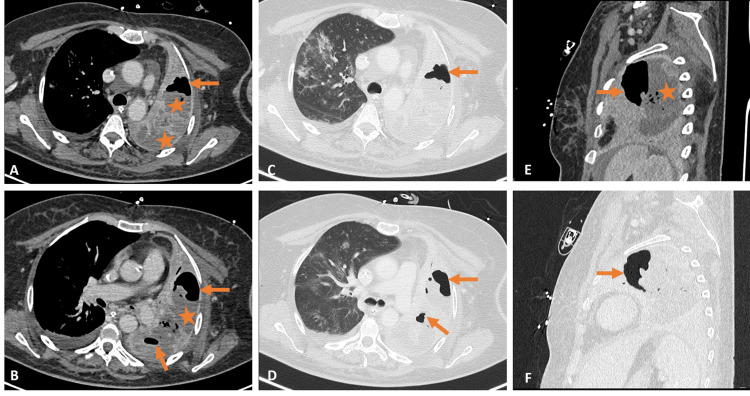
Chest computed tomography performed 17 days after hospitalization Axial enhanced computed tomography images at the apex (A) and mid-lung (B) continue to demonstrate heterogeneously enhancing left lung consolidation (asterisks) and increasing abscesses (arrows). Axial enhanced computed tomography images in lung windows at the apex (C) and mid-lung (D) and sagittal reformatted images in mediastinal (E) and lung (F) windows depict the same findings.

The case was discussed with the thoracic surgery team, confirming the need for surgical intervention but only after achieving clinical stabilization and control of the infectious focus. The clinical course remained unfavorable, with persistent distributive shock and increasing requirement for vasoactive support. The patient continued to receive poorly protective mechanical ventilation at volume control despite being on 6mL/Kg with lung protective ventilation strategy (Respiratory rate (RR)- 20, positive end-expiratory pressure (PEEP)- 14, tidal volume 6mL/Kg), the patient had plateau pressures of 40 cmH_2_O, driving pressures exceeding 22 cmH2O, and lung compliance below 20 mL/cmH_2_O. There was a rising trend in inflammatory parameters despite targeted antibiotic therapy, and the condition deteriorated further radiologically as lung abscesses increased in size based on a chest CT performed 17 days after admission (Figure 3). Ultimately, 20 days after admission, the patient’s clinical status remained unfavorable and irreversible.

## Discussion

We should consider NP in our differential diagnosis when pneumonia cases do not show favorable progression. It typically starts as a localized inflammatory process that advances to lung parenchymal destruction, leading to the development of cavities. Initially, affected areas exhibit capillary congestion within the alveoli, which increases the likelihood of thrombus formation and subsequent central vascular occlusion. This entire process disrupts the normal lung parenchymal structure, resulting in abscesses and cavities, where the penetration of antibiotic therapy becomes more challenging. The elevated morbidity and mortality associated with this condition stem not only from the causative agent but also from delayed diagnosis, typically occurring after an average of 9 days of illness. While S. pneumoniae is the most common culprit for this type of pneumonia, other microorganisms, such as S. aureus and S. viridans, have also been linked to pulmonary gangrene, albeit less frequently [3-5].

The process of parenchymal destruction is multifactorial, influenced not only by microorganism-specific factors but also host-related factors. The rapid buildup of capsular polysaccharides can overcome the cytolytic activity of alveolar macrophages due to their antigenic properties. Conversely, producing toxins like hemolysin and leukocidin further contributes to lung tissue damage. The addition of clindamycin is attributed to this latter factor, with documented benefits in reducing the release of pyrogenic substances and inducing membrane changes that render the bacteria more susceptible to the bactericidal activity of the immune system. Numerous host risk factors are described in the literature, such as male sex, advanced age, diabetes mellitus, chronic obstructive pulmonary disease, liver disease, heart failure, cerebrovascular disease, and other immunosuppressive states [2,4]. In a significant percentage of cases, the evolution is indolent, presenting clinically as an uncomplicated pneumonic condition. Laboratory studies often reveal leukocytosis, neutrophilia, thrombocytopenia, and elevated CRP. Its most common location is the right upper lobe [3,4].

The individual did not possess any of the mentioned risk factors in the case presented. Contrary to the more common clinical course, this patient experienced a rapid and unfavorable progression characterized by hemoptysis, hemodynamic instability, and severe hypoxemia, necessitating invasive mechanical ventilation. It is often observed that patients have been exposed to a course of antibiotics before hospital admission, but this was not the case here. Laboratory findings also showed a distinctive pattern compared to the cases described in the literature, notably displaying leukopenia and neutropenia. This atypical pattern may explain the comparatively less pronounced inflammatory response observed clinically. Pleural involvement may present as pleuritis, with pleural thickening up to the formation of a pleural effusion. In this case, on the second chest CT, a small collected pleural effusion was observed, corresponding to a complication of NP with a difficult approach from a medical point of view [2-4].

The diagnosis of NP depends on clinical and laboratory evaluation and imaging evaluation, with chest CT being the gold standard complementary diagnostic method. Serial radiographs are also extremely important for identifying disease progression. The initial empirical antibiotic therapy should be broad-spectrum, covering anaerobes. This typically involves the use of broad-spectrum penicillin or a 3rd or 4th generation cephalosporin in combination with clindamycin or metronidazole. Subsequent to microbiological isolation, targeted antibiotic therapy can be administered, as was done in the presented case. Bronchoscopy is a valuable diagnosis tool for complicated NP cases, such as those involving hemoptysis, empyema, or progressive lung destruction. However, in some cases, surgical intervention becomes necessary to achieve adequate infection control. The primary objective of surgery is to remove necrotic lung tissues, which are poorly permeated by antibiotics, and to address coexisting empyema [4,5].

In the case presented, there was a critical limitation in the level of treatment with a profound impact on prognosis. There is still controversy in the literature regarding the precise indications and the most appropriate time for surgery. A surgical approach is recommended in cases where there is a clinical deterioration or the presence of complications despite the adequate use of antimicrobials and less invasive procedures [6]. Some studies advocate performing the procedure while the disease is still localized, as delay in surgical intervention is associated with progressive infection in the lung parenchyma and higher rates of complications [6,7]. The surgical options in the literature are pleuropulmonary decortication, lobectomy, and closure of air leaks in patients with bronchopleural fistula through video-assisted thoracoscopic surgery (VATS). In the present case, the patient’s clinical instability, both hemodynamically and in terms of ventilation, rendered the escalation to a surgical approach unfeasible. In the opinion of the anesthetic and surgical team, the risk of death would be greater than the potential benefit.

Therefore, mechanical ventilation became progressively more unprotective, posing significant management challenges and limiting the medical team’s ability to minimize ventilator-induced lung injury. This subsequently impacted ventilation and oxygenation, ultimately leading to refractory shock. The unavailability of surgical intervention proved the decisive factor in the prognosis [4].

The analysis of this case underscores the significance of prompt diagnosis in managing critically ill patients. Swift diagnosis is pivotal for intensivists as it enables the stabilization of the patient, making surgical intervention technically feasible. Similar to the timely initiation of suitable antibiotic therapy, surgical intervention should not be postponed until a stage where, even with organ support, multiorgan dysfunction precludes an invasive approach to NP.

## Conclusions

NP should be considered in cases of CAP with a less favorable outcome. Early identification, medical therapy optimization, and timely recognition of the need for surgical therapy have a strong impact on morbidity and mortality. Although PNS presents high mortality, requiring a complex and challenging medical approach, a surgical approach is necessary for patients who do not respond to conservative treatment and may be associated with a better clinical outcome. Although carrying out prospective randomized studies is difficult given its rarity, research with several institutions and creating protocols could help in its better approach.
